# Increasing diagnostic accuracy to grade dysplasia in Barrett’s esophagus using an immunohistochemical panel for CDX2, p120ctn, c-Myc and Jagged1

**DOI:** 10.1186/s13000-016-0473-7

**Published:** 2016-02-29

**Authors:** Dipti M. Karamchandani, Heather L. Lehman, Sara E. Ohanessian, Julie Massé, Patricia A. Welsh, Robert D. Odze, John R. Goldblum, Arthur S. Berg, Douglas B. Stairs

**Affiliations:** Department of Pathology, The Pennsylvania State University College of Medicine, Penn State Milton S. Hershey Medical Center, 500 University Drive, Hershey, PA 17033 USA; Department of Pathology, Brigham and Women’s Hospital, Boston, MA USA; Department of Pathology, Cleveland Clinic, Cleveland, OH USA; Department of Public Health Sciences, The Pennsylvania State University College of Medicine, Hershey, PA USA

**Keywords:** Barrett’s esophagus, Dysplasia, Low-grade, High-grade, Esophageal adenocarcinoma, Immunohistochemistry, Biomarkers

## Abstract

**Background:**

Patients with non-dysplastic Barrett’s esophagus (ND-BE) and low-grade dysplasia (LGD) are typically monitored by periodic endoscopic surveillance, while those with high-grade dysplasia (HGD) and esophageal adenocarcinoma (EAC) are usually treated by more aggressive interventions like endoscopic mucosal resection, ablation or surgery. Therefore, the accurate grading of dysplasia in Barrett’s esophagus (BE) is essential for proper patient care. However, there is significant interobserver and intraobserver variability in the histologic grading of BE dysplasia. The objective of this study was to create an immunohistochemical (IHC) panel that facilitates the grading of BE dysplasia and can be used as an adjunct to histology in challenging cases.

**Methods:**

100 BE biopsies were re-graded for dysplasia independently by 3 subspecialized gastrointestinal pathologists. IHC staining for CDX2, p120ctn, c-Myc and Jagged1 proteins was then performed and assessed by two separate methods of semi-quantitative scoring. Scores were integrated using a principal component analysis (PCA) and receiver operating characteristic (ROC) curve.

**Results:**

Principal component analysis demonstrated the ability of this panel of proteins to segregate ND-BE/LGD and HGD/EAC, as the expression of the four proteins is significantly altered between the two subsets. Analysis of the receiver operating characteristic curve showed that this panel has the potential to aid in the grading of dysplasia in these two subcategories with both high sensitivity and specificity. While not able to discriminate between ND-BE and LGD, this panel of four proteins may be used as an adjunct to help discriminate subsets of ND-BE/LGD from HGD/EAC.

**Conclusions:**

We propose that the maximum utility of this IHC panel of CDX2, p120ctn, c-Myc, and Jagged1 proteins would be to distinguish between LGD and HGD in histologically challenging cases, given the aggressive interventions still used for HGD in many institutions, and hence may aid in the optimal patient management. The results of this initial study are promising, though further validation is needed before this panel can be used clinically, including future randomized prospective studies with larger patient cohorts from diverse locations.

**Electronic supplementary material:**

The online version of this article (doi:10.1186/s13000-016-0473-7) contains supplementary material, which is available to authorized users.

## Background

Esophageal adenocarcinoma (EAC) is the predominant form of esophageal cancer in the United States [1] and incidence of EAC has experienced the highest rate of increase among all solid tumors during the past 30 years [2, 3]. EAC is typically diagnosed at a late stage of the disease, and therefore, has a poor 5-year survival rate of approximately 20 %. Barrett’s esophagus (BE) is a known precursor lesion for EAC [4] and at present, the best predictive factor for the future development of EAC is the identification of BE with high-grade dysplasia (HGD).

The progression of BE through a metaplasia-dysplasia-adenocarcinoma sequence is slow and unpredictable. The rate of progression of patients with low-grade dysplasia (LGD) historically was thought to be close to that of ND-BE, under 1 %. However, more recent studies have suggested a higher rate of progression for LGD (2–28 % per year) [5–10]. Furthermore, HGD has a high rate of progression to EAC (6–60 % per year) [6, 10, 11]. Currently, in many institutions, detection of non-dysplastic BE (ND-BE) and BE with LGD necessitates periodic endoscopic surveillance, whereas HGD and EAC warrants more aggressive interventions like endoscopic resection, ablation and/or esophagectomy [12, 13]. Therefore, accurate grading of dysplasia, and one could argue more so accurate diagnosis of HGD/EAC, is crucial for optimal patient management. However, grading BE dysplasia can be challenging, and in fact, a recent multi-institution international study has documented that HGD in BE is overdiagnosed in about 40 % of the cases, which can lead to possible unwarranted therapy and mismanagement, including unnecessary esophagectomy, in some patients [14]. Currently, histologic assessment of BE biopsies remains the gold standard for risk assessment [15]. Though, histological examination does get complicated by significant interobserver and intraobserver variability [5, 16, 17]. Thus, there is a need for adjunct markers that can be used to assist histologic assessment in rendering a more accurate diagnosis, especially in diagnostically challenging cases, and probably more so for an accurate diagnosis of HGD/EAC in BE, given the treatment implications.

In this study, we analyzed the expression of CDX2, p120-catenin (p120ctn), c-Myc and Jagged1 proteins and assessed the value of the combination of these four proteins to aid in accurately grading dysplasia in BE biopsy samples. We selected proteins that are involved in independent signaling pathways and are differentially regulated during the progression from ND-BE to EAC [18, 19]. CDX2 is involved in initiation of transdifferentiation of normal esophageal squamous epithelium into columnar epithelium [20]. Its expression is high in ND-BE and progressively decreases in dysplasia and EAC [21]. p120ctn is a tumor suppressor gene. Prior studies have shown that its expression is decreased in 20 % of ND-BE and 70 % of EAC, compared to normal esophageal squamous epithelium [22]. In contrast, c-Myc is an oncogene that is upregulated in greater than 70 % of EAC [6, 23]. Finally, Jagged1 is a Notch canonical pathway member and is downregulated in various cancers, including EAC [1, 2, 21, 24].

Discrimination between ND-BE, LGD, HGD and EAC is important in determining appropriate patient care. To this end, the purpose of this study was to develop an ancillary immunohistochemical panel that can be combined with histological analysis to facilitate the discrimination of ND-BE, LGD, HGD, and EAC and help to more accurately grade BE dysplasia, especially in histologically challenging cases. In this initial study we analyzed a cohort of 100 BE biopsy samples with varying grades of dysplasia for differential expression of these four proteins by IHC.

## Methods

### Cases

This research was approved by the Hershey Medical Center Institutional Review Board. A Health Insurance Portability and Accountability Act (HIPAA) compliant retrospective review of surgical pathology files at the Hershey Medical Center was performed. The pathology database was searched for ND-BE, BE with LGD, HGD and EAC cases (biopsies and endoscopic mucosal resections) diagnosed between 2001 and 2014 with available formalin-fixed, paraffin embedded tissue blocks. Cases diagnosed as BE with epithelial alterations indefinite for dysplasia were excluded from this study. A total of 100 samples were retrieved and Hematoxylin and Eosin (H&E)-stained slides were re-reviewed independently by three subspecialized academic gastrointestinal (GI) pathologists at three different institutions. All patients had Barrett’s esophagus as defined by the American College of Gastroenterology ^3^, which includes both histologic (intestinal metaplasia with goblet cells) and endoscopic (columnar-type mucosa) components [13]. The cases were classified as ND-BE, BE with LGD, BE with HGD and EAC (including intramucosal and submucosal EAC) independently by these three GI pathologists using previously published diagnostic criteria [16, 25–27]. In brief, the criteria were as follows:

#### Negative for dysplasia

The architecture is within normal limits. Although nuclear hyperchromasia, stratification and elongation can be seen in the regenerative compartment, there is complete surface maturation. The nuclei are arranged in a uniform surface monolayer and do not vary greatly in size or shape.

#### LGD

Nuclear hyperchromasia, elongation and stratification is seen in the basal compartment and these changes extend to the surface epithelium. However, nuclear polarity is maintained in LGD. Mild architectural complexity with glandular crowding may be seen.

#### HGD

The biopsies showed severe cytologic atypia extending to the surface epithelium, defined, among others, by loss of nuclear polarity. Other cytologic features such as marked nuclear enlargement, pleomorphism, hyperchromatism, and irregular nuclear membranes are also present in HGD. Architectural features, such as glandular complexity and distortion with crowding and dilated glands with intraluminal necrotic debris, are also present.

#### Intramucosal EAC (IMC)

The diagnosis of IMC entails either single cell invasion of lamina propria by neoplastic cells or abortive, angulated glands infiltrating the lamina propria. The other criteria include sheets of cells obliterating the lamina propria and a never-ending/anastomosing gland pattern.

#### Submucosal EAC (SMC)

SMC requires unequivocal stromal desmoplasia to be present in the biopsies.

### Immunohistochemistry

IHC analysis was performed on 5 μm sections for CDX2, p120ctn, c-Myc and Jagged1 proteins. Tissue sections were baked 1 h at 55 °C, deparaffinized with xylene and antigens were unmasked by heating in citrate buffer (0.01 M, pH 6.0). Endogenous peroxidase activity was blocked by incubation with 3 % peroxide for 6 min. The slides were incubated overnight at 4 °C with primary antibodies (CDX2: Biogenex, Fremont, CA, # Mu392A-UC, dilution 1:50; p120ctn: BD Biosciences, San Jose, CA, # 610134, dilution 1:100; c-Myc: Epitomics Inc., Burlingame, CA, # 1472-1, dilution 1:100). For antibody detection, ImmPRESS (Vector Labs, Burlingame, CA) anti-rabbit or anti-mouse antibodies were used. Slides were incubated with DAB for 10 min and counterstained with hematoxylin prior to coverslipping with Permount. For Jagged1 and p53 staining, the Penn State Hershey Molecular and Histopathology Core Research Lab performed IHC on the GE Discovery XT stainer, using an EDTA based retrieval solution (Ventana Medical Systems, Tucson, AZ). The slides were incubated with Jagged1 antibody at a 1:400 dilution (Jagged1: Sigma Aldrich, Saint Louis, MO, #HPA021555) and p53 was a ready to use antibody from Ventana Medical Systems (#790-2912).

### IHC quantification

Immunohistochemical staining was evaluated on an Olympus BX53 light microscope at 100×, 200× and 400× magnifications and images were captured using an Olympus DP25 camera and Olympus CellSens Dimension software. All the immunohistochemical slides were semi-quantitatively analyzed and interpreted without knowledge of the histologic diagnosis by one person in order to eliminate interobserver bias in interpretation of the immunohistochemical results. These slides were analyzed at 200× using two different methods as follows:

#### Quartile scoring method

Score of 0 = no staining; 1 = 1 % to 25 % positive; 2 = 26 % to 50 % positive; 3 = 51 % to 75 % positive; 4 = 76 % to 100 % positive.

#### IRS scoring method

An immunoreactivity score (IRS) was utilized as previously described [24, 28]. IRS values are calculated as the staining intensity value (0 to 3) multiplied by the estimated value of the percentage of positively stained cells. Percentage of stained cells was scored as: 1 = 10 % to 25 % positive; 2 = 26 % to 50 % positive; 3 = 51 % to 75 % positive; 4 = 76 % to 100 % positive. Intensity was scored as: 0 = no staining; 1 = weak staining; 2 = moderate staining; 3 = strong staining. If less than 10 % of the cells were stained, a score of zero was given. Therefore, the total IRS ranged from 0 to 12.

While the quartile scoring method is currently the more standard method used by pathologists, the IRS scoring method is a standard research tool and its utility has been validated in multiple prior studies ^24,28^. Therefore, both semi-quantitative methods were evaluated to determine the best method in grading and diagnosing dysplasia in BE.

### Statistical analysis

Samples were classified into two groups, ND-BE/LGD and HGD/EAC based on histologic diagnosis and compared to each other using the Student’s t-test. Analysis was also performed to see if this panel could facilitate discrimination of ND-BE biopsies from LGD/HGD/EAC. Semi-quantitative scoring and their subsequent analysis by principal component analysis (PCA) and receiver operating characteristic (ROC) curve were performed. PCA was applied to the four IHC staining scores to reduce the dimension to two principal components. The two principal components were graphed and colored according to the clinical status (ND-BE/LGD and HGD/EAC) of the patients. The first principal component versus clinical status was separately graphed as a boxplot. To evaluate the utility of the potential marker, a ROC curve was constructed to assess the prediction ability to identify ND-BE/LGD and HGD/EAC patients using the scores of the immunostaining for the four proteins. All tests were carried out at a significance level of 0.05. The statistical analysis was performed using R version 3.0.0 (http://www.r-project.org).

## Results

### Patient characteristics

100 patient samples were included in the study. The initial diagnoses were ND-BE (*n* = 24), LGD (*n* = 23), HGD (*n* = 24) and EAC (*n* = 29). The age of the patients ranged from 31 to 89 years (mean 64.4, median 65) with a male-to-female ratio of 4:1 (79 men and 21 women).

### Histologic assessment of samples

**Fig. 1:**
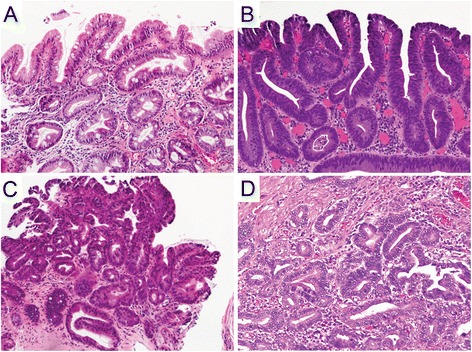
Hematoxylin & Eosin staining. **a** Non-dysplastic Barrett’s mucosa characterized by uniform, bland nuclei arranged in a surface monolayer. **b** Low-grade dysplasia exhibiting nuclear hyperchromasia, elongation and stratification extending up to the surface epithelium. **c** High-grade dysplasia depicting increased architectural and cytologic complexity including loss of nuclear polarity. **d** Intramucosal adenocarcinoma characterized by severe architectural distortion including angulated glands. (a-d, 100×)

Three subspecialized GI pathologists re-reviewed and re-graded the 100-sample cohort independently using criteria outlined above. Upon completion of histologic assessment, the cohort was divided into:

1. Complete consensus: All three pathologists made the same diagnosis independently in 62 of 100 samples (62 %). This consisted of 26 ND-BE, 6 LGD, 9 HGD and 21 EAC (Fig. 1a-d).

**Only samples with a complete consensus diagnosis were included in the initial statistical analyses** (See “Statistical analyses of scoring methods using complete consensus cases” section below).

2. Partial consensus: Two of three pathologists were in agreement and one was in disagreement in 37 samples (37/100 samples; 37 %). No individual pathologist was in disagreement more than the other pathologists, suggesting that these partial consensus samples may represent diagnostically challenging cases. These partial consensus cases were as follows:
  A. Partial consensus between BE-ND and LGD - 10/37 cases.
  B. Partial consensus between LGD and HGD - 7/37 cases.
  C. Partial consensus between BE-ND and HGD - 1/37 cases. This case was diagnosed as ND-BE by two pathologists and as HGD by the third.
  D. Partial consensus between HGD and EAC - 19/37 cases.

3. Non-consensus: All three pathologists diagnosed one sample differently. It was diagnosed as LGD by the first, HGD by the second and EAC by the third (1/100; 1 %).

**Partial and non-consensus samples were examined secondarily to see how they overlaid with consensus samples** (See “Statistical analyses of scoring methods using partial/non-consensus cases” section below).

### Immunohistochemical staining

#### CDX2

**Fig. 2:**
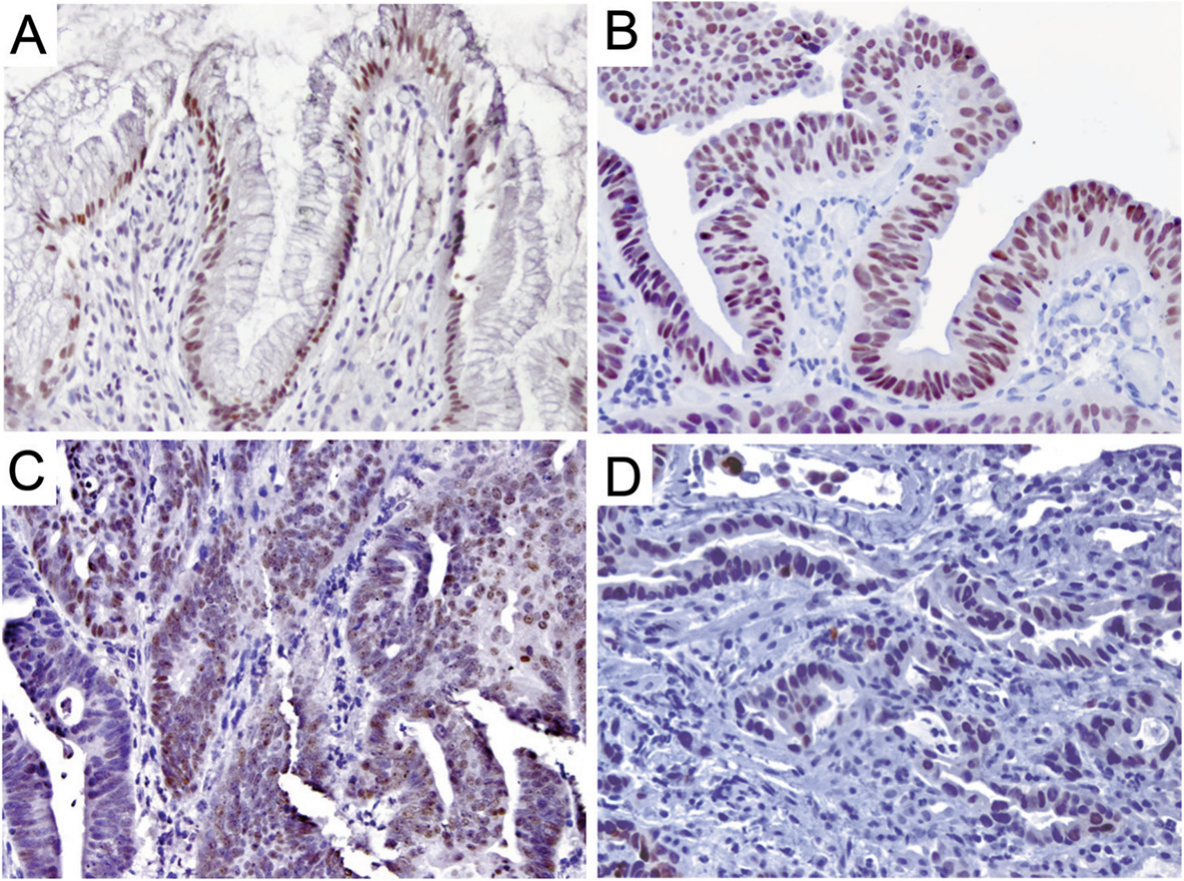
CDX2 staining pattern in Barrett’s esophagus. **a, b** Non-dysplastic BE & Low-grade dysplasia, respectively, depicting diffuse nuclear staining. **c, d** High-grade dysplasia & adenocarcinoma, respectively, depicting a decrease in nuclear intensity as well as percentage of positive cells. (a&b, 200×; c&d, 400×)

Expression of CDX2 decreased significantly through the progression from ND-BE to EAC (Fig. 2). Nuclear CDX2 staining was present in the majority of glandular cells in ND-BE and LGD samples, with an average score of 3.4 in ND-BE samples and 3.3 in LGD samples, using the quartile scoring method (Fig. 2a and b). The percentage of cells stained with CDX2 was decreased in HGD and EAC samples, with an average score of 2.7 in both HGD and EAC samples.

In HGD and EAC samples, nuclear CDX2 staining was also decreased in intensity, with an average IRS score of 4.2 in HGD and 4.1 in EAC samples compared to an average IRS score of 7.0 in ND-BE and 6.2 in LGD samples (Fig. 2c and d). Average scores for CDX2 staining using the quartile and IRS scoring methods are presented in Table 1. ANOVA analysis of CDX2 scores revealed a significant decrease in expression of CDX2 expression as ND-BE progressed to EAC (*p* = 0.01 for quartile scoring, *p* ≤ 0.01 for IRS scoring) (Additional file 1: Figure S1a and b).

**Table 1:** Mean values of IHC scores for CDX2, p120ctn, c-Myc and Jagged1

	CDX2	Membranous p120ctn	Cytoplasmic p120ctn	c-Myc	JAG-1
Quartile scoring method					
ND-BE	3.4	3.7	0.6	2.2	3.6
LGD	3.3	4.0	0.7	2.2	3.5
HGD	2.7	3.6	2.1	2.8	3.8
EAC	2.7	2.8	3.0	2.7	3.9
* p*-value	*p* ≤ 0.01	*p* ≤ 0.001	*p* < 0.0001	*p* = 0.17	*p* = 0.10
IRS scoring method					
ND-BE	7.0	8.0	0.6	2.8	4.3
LGD	6.2	10.0	0.8	3.2	5.2
HGD	4.2	6.2	2.4	5.2	5.3
EAC	4.1	4.1	3.0	4.6	6.6
* p*-value	*p* ≤ 0.01	*p* ≤ 0.001	*p* ≤ 0.001	*p* ≤ 0.05	*p* ≤ 0.001

#### p120ctn

**Fig. 3:**
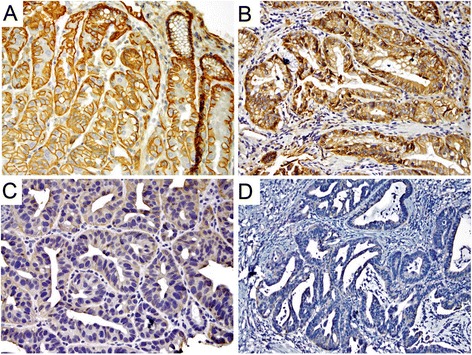
p120ctn staining pattern in Barrett’s esophagus. **a** Non-dysplastic BE depicting a strong and well-defined membranous expression pattern in the columnar epithelial cells. **b** Low-grade dysplasia depicting a membranous expression as well as cytoplasmic mislocalization in some cells. **c, d** High-grade dysplasia & adenocarcinoma, respectively, showing a significant decrease in membranous staining and partial cytoplasmic mislocalization in neoplastic cells. (a&c, 400×; b&d, 200×)

p120ctn had a strong and well-defined expression pattern at the membrane of the columnar epithelial cells of ND-BE samples (Fig. 3a). For membranous p120ctn expression, ND-BE samples had an average score of 3.7 using the quartile scoring method and an IRS score of 8.0. In comparison, cytoplasmic p120ctn scores were on average 0.6 for the quartile scoring method and 0.6 for IRS scoring. LGD samples showed expression of p120ctn at the membrane as well as mislocalization to the cytoplasm of some cells (Fig. 3b). p120ctn membranous expression was scored as 4 with the quartile scoring method and the IRS score was 10.0 in LGD samples. Cytoplasmic p120ctn scores were lower, with an average quartile method score of 0.7 and an IRS score of 0.8. p120ctn membranous expression was significantly decreased in HGD and EAC samples and the protein was partially mislocalized to the cytoplasm in a larger percentage of cells (Fig. 3c and d). Membranous p120ctn staining scores were 3.6 for HGD and 2.8 for EAC samples with the quartile scoring method. IRS scores were significantly lower as well; 6.2 for HGD and 4.1 for EAC. Conversely, cytoplasmic p120ctn significantly increased in HGD and EAC samples compared with ND-BE and LGD samples. Cytoplasmic p120ctn staining scores were 2.1 for HGD and 3.0 for EAC using the quartile scoring method. IRS scores were 2.4 for HGD and 3.0 for EAC. Average scores of the membranous and cytoplasmic p120ctn staining are summarized in Table 1. These data demonstrate that through the progression from ND-BE to EAC, p120ctn membranous expression is significantly decreased (*p* ≤ 0.001 for both scoring methods) (Additional file 1: Figure S1c and d). Conversely, p120ctn cytoplasmic expression is significantly increased through disease progression (quartile scoring method, *p* < 0.0001; IRS scoring, *p* ≤ 0.001) (Additional file 1: Figure S1e and f).

#### c-Myc

**Fig. 4:**
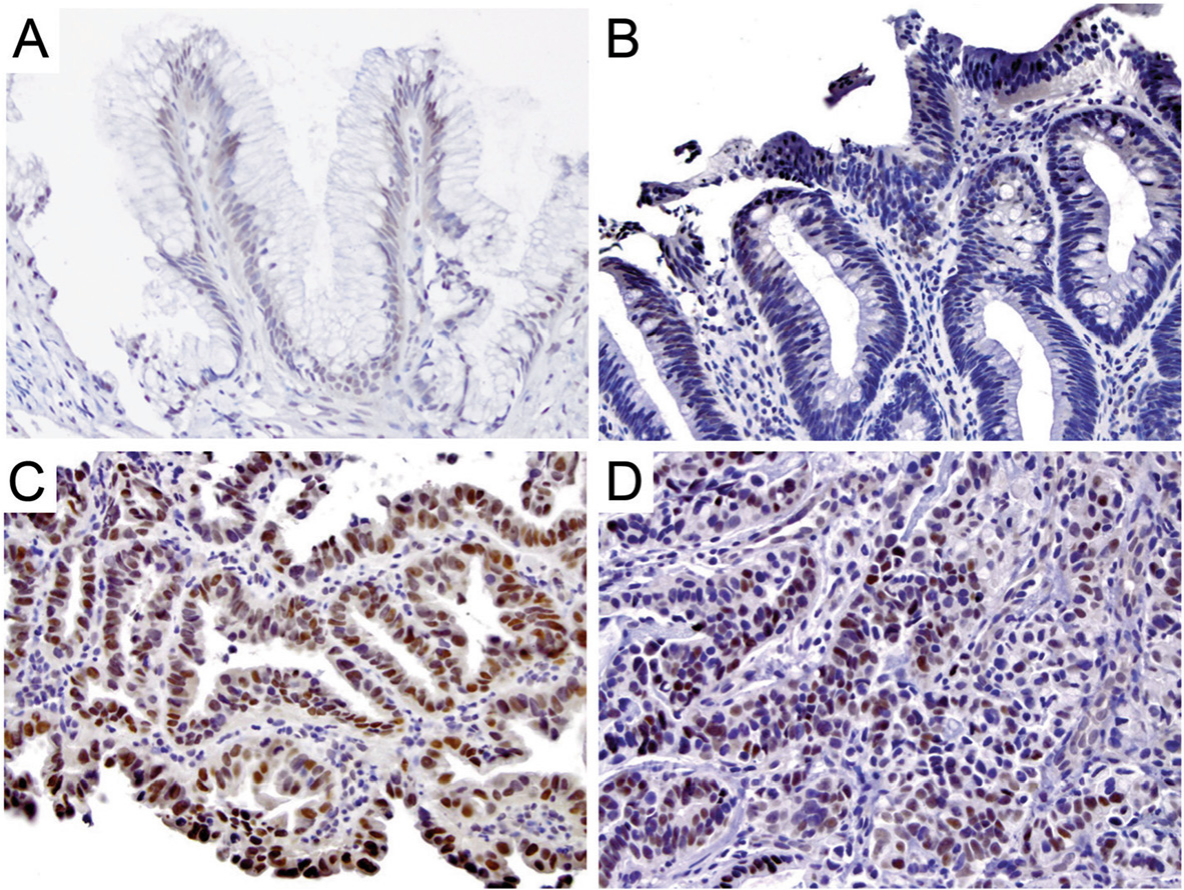
c-Myc staining pattern in Barrett’s esophagus. **a, b** Non-dysplastic BE & Low-grade dysplasia, respectively showing weak nuclear expression. **c, d** High-grade dysplasia & adenocarcinoma, respectively, depicting a stronger nuclear expression. (a&b, 200×; c&d, 400×)

Immunohistochemical staining showed weak nuclear expression for c-Myc in ND-BE and LGD samples, with a lower percentage of positively stained cells compared to HGD and EAC. IHC scoring of ND-BE c-Myc staining yielded an average scores of 2.2 with the quartile scoring method and an average IRS score of 2.8 (Fig. 4a). LGD samples had an average quartile score of 2.2 and an IRS score of 3.2 (Fig. 4b). In contrast, HGD and EAC samples had significantly stronger nuclear c-Myc expression (Fig. 4c and d). The quartile scoring method resulted in average staining scores of 2.8 for HGD and 2.7 for EAC, indicating an increased percentage of positive cells when compared to ND-BE and LGD. However, IRS scoring resulted in a significantly higher score of 5.2 for HGD samples and 4.6 for EAC when compared to ND-BE and LGD. Means of the c-Myc staining scores are summarized in Table 1. These data suggest that nuclear c-Myc expression increases during the progression of the disease. Although an increase in score was seen by both scoring methods, the results were statistically significant only by IRS scoring (quartile scoring method, *p* = 0.17; IRS scoring, *p* = 0.02) (Additional file 1: Figure S1g and h).

#### Jagged1

**Fig. 5:**
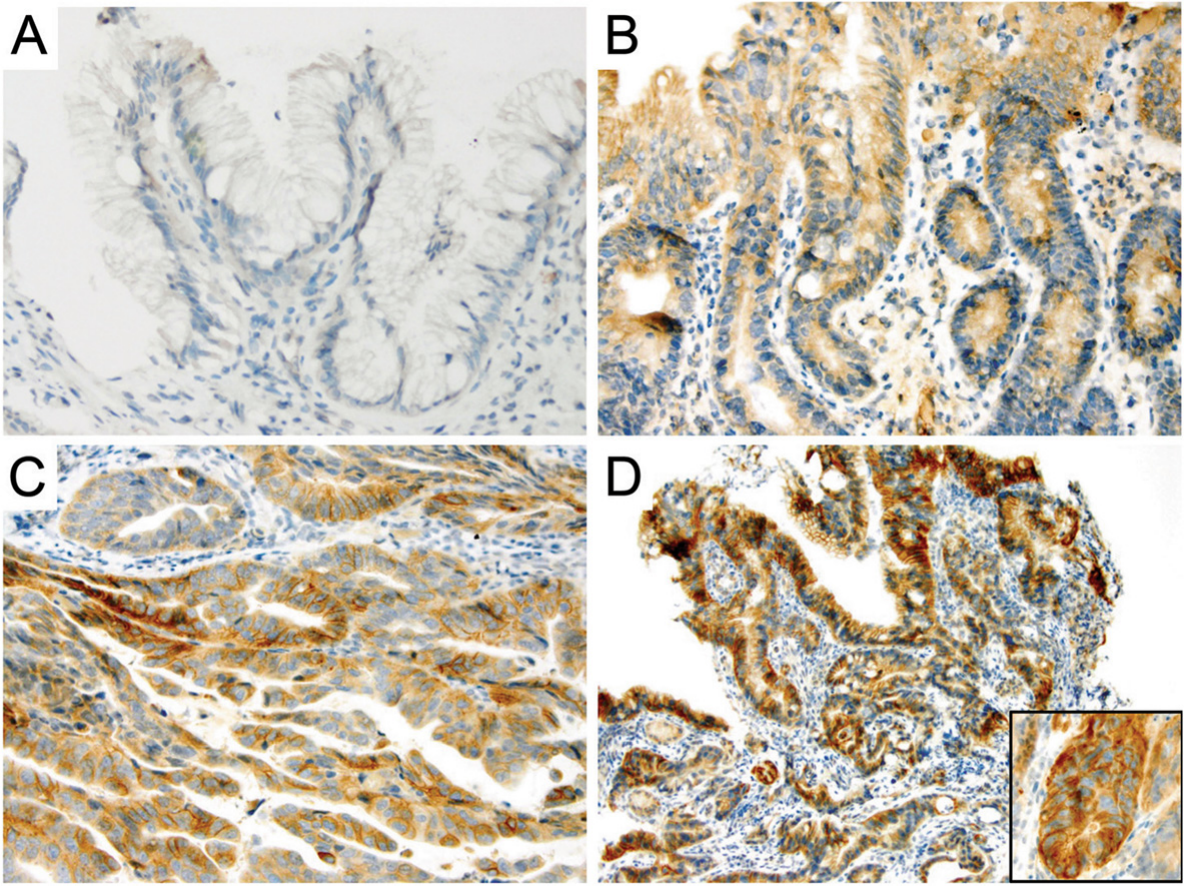
Jagged1 staining pattern in Barrett’s esophagus. **a** Non-dysplastic Barrett’s mucosa showing faint membranous expression. **b** Low-grade dysplasia showing membranous localization along with weak diffuse cytoplasmic staining. **c, d** High-grade dysplasia & adenocarcinoma, respectively, showing diffuse staining with moderate membranous and cytoplasmic expression. The inset in panel (**d**) further highlights this staining pattern and staining intensity. (a&d, 200×; b&c, and d-inset 400×)

Jagged1 had weak membranous staining of columnar cells in ND-BE samples and a staining score of 3.6 using the quartile scoring method. ND-BE samples yielded an IRS score of 4.3 (Fig. 5a). The same localization was observed in LGD samples but with slightly diffuse cytoplasmic staining (Fig. 5b). Using the quartile scoring method the score for LGD samples was 3.5 and the IRS score was 5.2. In HGD and EAC samples, Jagged1 was detected in a large proportion of cells and appeared to be highly diffuse with moderate membranous and cytoplasmic expression (Fig. 5c and d). Using the quartile scoring method the score for HGD was 3.8 and EAC was 3.9. While the quartile scoring method did not show a statistically significant change in Jagged1 staining (*p* = 0.10), IRS scoring of membranous Jagged1 expression showed a significant increase in expression from ND-BE/LGD (IRS score 4.8) to HGD/EAC (IRS score 6.0) patient samples (*p* ≤ 0.001) (Additional file 1: Figure S1i and j). Also, Jagged1 was significantly higher in HGD compared with LGD when analyzed using IRS scoring, illustrating a potential switch of expression occurring between these two pathologic states. Means of the Jagged1 staining scores using two scoring methods are presented in Table 1.

### Statistical analyses of scoring methods using complete consensus cases

Only samples with a complete consensus diagnosis were included in initial statistical analyses (i.e. 62 of 100 samples analyzed). To determine the ability of these four markers to classify varying grades of neoplasia in BE, we utilized the scores obtained from the quantification of each of the four proteins together using two individual scoring methods described above. We rigorously analyzed the ability of the four-marker panel to distinguish between ND-BE, LGD, HGD, or EAC in any manner. Unsupervised PCA analysis of the two scoring methods looked at whether the four proteins could aid in classifying ND-BE from LGD/HGD/EAC. Our analysis demonstrated that the markers were not able to separate ND-BE samples from the combined LGD/HGD/EAC samples as a group.(*p* ≥ 0.05; data not shown).

**Fig. 6:**
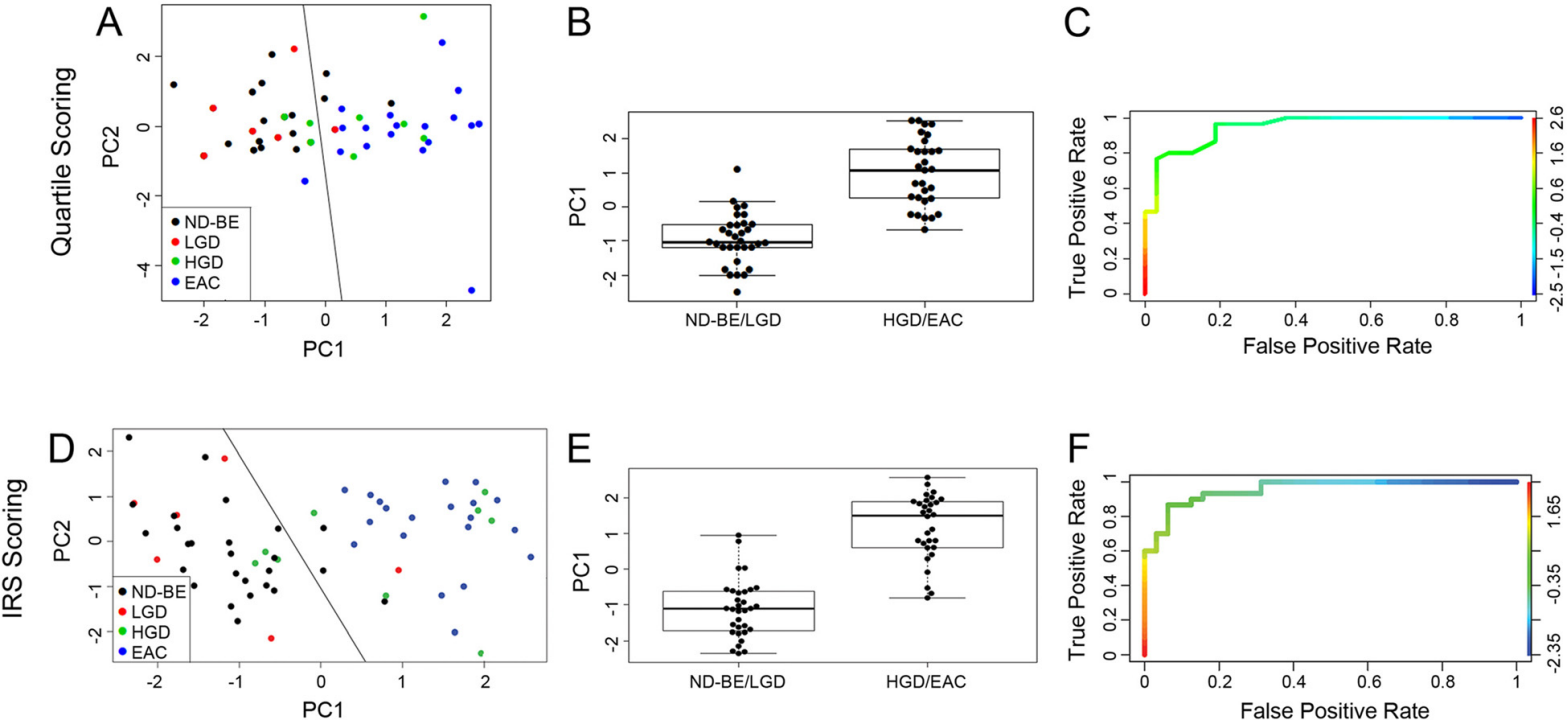
Statistical analysis of IHC quantification using complete consensus samples. **a** Principal component analysis of quartile scoring method scores was generated from ND-BE (black circles), LGD (red circles), HGD (green circles) and EAC (blue circles) consensus samples. A demarcation line was added for easy visualization. **b** Beeswarm plot representing the distribution of ND-BE/LGD and HGD/EAC consensus samples when scored with the quartile scoring method. **c** Receiving operating characteristic curve depicting the accuracy of CDX2, p120ctn, c-Myc and Jagged1 expression scores as markers of diagnosis with a confidence interval of 95 % for the quartile scoring method. The area under curve for this ROC was 0.866. **d** PCA of IRS scores using IRS scoring, with circles representative of disease state as noted in (**a**). **e** Beeswarm plot when scored with the IRS scoring method (*p* ≤ 0.001). **f** ROC curve when scored with IRS scoring. The area under curve for this ROC was 0.956

The greatest changes in expression of the four markers were detected between LGD and HGD. For the quartile scoring method, where a score of zero indicates no staining and scores 1 through 4 are divided into quartiles of percentage of cells stained, an unsupervised PCA was able to segregate samples into two groups – ND-BE/LGD and HGD/EAC patients (Fig. 6a). This observation was confirmed by the box plot graph of the first principal component, which showed a significant change in the distribution of the samples over the two groups (*p* ≤ 0.001) (Fig. 6b). A ROC curve was also plotted to determine the ability of the integrated IHC scores for the four proteins to distinguish ND-BE/LGD from HGD/EAC. ROC analysis provided 81 % sensitivity and 83 % specificity, with area under the curve of 0.866 (Fig. 6c).

An unsupervised PCA on the IRS scoring method, where a score incorporates both the percentage of cells stained and intensity of the stain, was also able to segregate samples into ND-BE/LGD and HGD/EAC groups (Fig. 6d). The box plot graph of the first principal component showed a significant change in the distribution of the samples over the two groups (*p* ≤ 0.001) (Fig. 6e). The ROC curve analysis provided 87 % sensitivity and 87 % specificity and area under the curve of 0.956 (Fig. 6f).

Upon examining sensitivity, specificity, and area under the curve, these data suggest that although both scoring methods were able to separate the samples into two subcategories, the IRS scoring method results in higher sensitivity and specificity and a lower area under the curve. Therefore, the IRS scoring method is the better and more accurate method to segregate ND-BE/LGD and HGD/EAC patients.

### Statistical analyses of scoring methods using partial/non-consensus cases

**Fig. 7:**
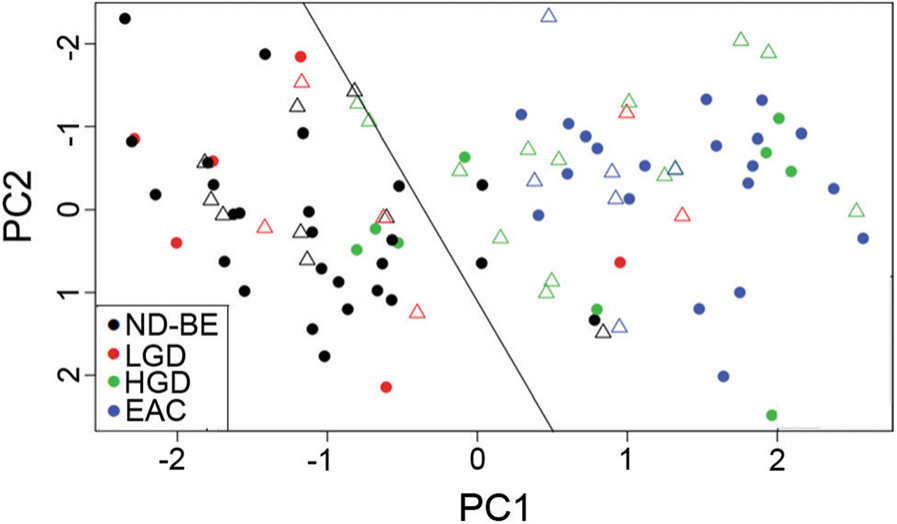
Statistical analysis of IHC quantification using partial/non-consensus samples. Non-consensus samples overlaid onto the PCA analysis of the consensus samples when scored with the IRS scoring method. ND-BE (black triangles), LGD (red triangles), HGD (green triangles) and EAC (blue triangles) are non-consensus samples

Although the quartile scoring method is currently the more conventional method used by pathologists, this scoring method was excluded for analysis of the partial/non-consensus patient samples in this sentinel study since it did not perform as well as the IRS scoring method. PCA analysis of the quartile scoring method demonstrated considerable overlap of ND-BE and HGD samples as well as lower sensitivity, specificity, and ROC area under the curve values. For these reasons, we focused on IRS scoring to test if our IHC panel can aid in the accurate diagnosis of partial/non-consensus samples. We applied the PCA analysis of the consensus samples to the 38 partial/non-consensus samples to examine if the parameters used to segregate complete consensus samples would also be able to segregate partial/non-consensus samples. This was completed for the IRS scoring method, as ROC curve analysis and area under the curve demonstrated that the IRS scoring method is the best predictive scoring method for BE dysplasia classification. These results are displayed as a graph of principal component 1 versus principal component 2 (Fig. 7).

These hybrid PCAs demonstrate that a significant proportion of the partial consensus samples also segregate based on the majority diagnosis of the sample by two of the three expert GI pathologists. For IRS scoring, seven of nine partial consensus ND-BE samples were correctly clustered into the consensus ND-BE/LGD group while four of six partial consensus LGD samples clustered correctly. All partial consensus HGD and EAC samples clustered with the consensus HGD/EAC cases. The one non-consensus case (diagnosed as LGD by one GI pathologist, HGD by the second and EAC by the third) overlaid with the complete consensus HGD/EAC cases. Nonetheless, ROC curve analysis demonstrated 89 % sensitivity and 90 % specificity and area under the curve of 0.948 irrespective of the consensus status of the samples (data not shown). These data suggest that IRS scoring, which takes into account both the percentage of cells stained as well as the intensity of the stain, can better predict the diagnosis of diagnostically challenging cases.

Partial consensus cases may represent interobserver and intraobserver variability, diagnostically challenging cases, or a combination thereof. All partial consensus cases (37 samples; 1 non-consensus sample was excluded) were re-evaluated based on pathology by the three subspecialized GI pathologists. Specifically, the pathologist with the non-consensus interpretation in each case re-read the respective slides having been supplied the interpretations of all three pathologists. Reinterpretations of the partial consenus samples demonstrated that the pathologists changed their diagnoses in 21 of 37 (57 %) samples (data not shown). Upon analysis of the changes in diagnosis, no new discernable patterns or associations emerged in relation to the diagnostic categories or individual pathologists. Given that there was no significant shift in diagnoses after reinterpretation, we believe this indicates that these cases are in fact diagnostically challenging, and further strengthens the value and relevance of our proposed IHC panel for use in diagnostically challenging cases of BE dysplasia.

### Tp53 expression as a marker of LGD progression

As mentioned previously, two of the six partial consensus LGD samples clustered with the consensus HGD/EAC cases upon PCA analysis. Since the majority of the partial consensus samples overlap closely with the consensus samples, this allows us to further analyze any outliers. We questioned whether the partial consensus LGD samples that clustered with HGD/EAC cases (which we have termed “outliers”) (Fig. 7), would represent disease that would be more likely to progress to HGD/EAC. To test this we investigated the Tp53 status of the six partial consensus LGD samples, as Tp53 is increasingly being implemented as a marker of LGD progression [29, 30]. IHC analysis of Tp53 demonstrated that two of the six partial consensus LGD samples had positive staining. Of the two positive samples, one was an outlier that had clustered with the HGD/EAC samples (Additional file 2: Figure S2). These data demonstrate that there is no relationship between the LGD outlier samples and Tp53 status, and therefore, Tp53 analysis does not complement our proposed four-marker IHC panel.

## Discussion

Barrett’s esophagus is the predominant risk factor for EAC, the most prevalent form of esophageal cancer in the United States. The accurate grading of dysplasia in BE is subject to significant interobserver and intraobserver variability, and this has been confirmed by multiple prior studies [5, 16, 17]. Notably in this study, three subspecialized GI pathologists independently and blindly re-graded dysplasia in 100 BE biopsies. Despite using the same diagnostic criteria outlined above, the three GI pathologists achieved partial or no consensus in a substantial number of cases (38 %). Noticeably, no single pathologist was in disagreement more than the other pathologists in the partial consensus cases, proving that some of these indeed are diagnostically challenging cases. Accurate grading of dysplasia and especially discrimination of LGD from HGD is important because of the aggressive treatment interventions still used for HGD in many institutions. A recent multicenter international study concluded that HGD in BE is overdiagnosed in about 40 % of the cases, which can lead to possible unwarranted therapy and grave mismanagement, including unnecessary esophagectomy, in some patients [14]. All these data reemphasize the variability that exists when diagnosing dysplasia in BE, even amongst specialized GI pathologists, and the need for a diagnostic adjunct to help more accurately grade BE dysplasia, especially in diagnostically challenging cases.

No reliable diagnostic markers are currently available to aid in the diagnosis of BE, which is based on histopathologic examination of biopsies. In this study, we analyzed the expression of four proteins from different signaling pathways (CDX2, p120ctn, c-Myc and Jagged1) in the progression of ND-BE to EAC, with the objective to increase the diagnostic accuracy of grading neoplasia in BE. We initially focused our analyses on the 62 samples that had complete diagnostic consensus between three independent pathologists. Partial/non-consensus samples were examined secondarily. We found that in cases in which there was a complete consensus, regardless of the scoring method used, nuclear CDX2 and p120ctn expression were both significantly decreased between ND-BE/LGD and HGD/EAC samples, whereas c-Myc and Jagged1 expression were both upregulated in the HGD/EAC groups. PCA analysis that integrates the score of each of these four proteins illustrates that a combinatorial protein expression analysis approach can segregate the patients into two broad categories (ND-BE/LGD and HGD/EAC) by both quartile and IRS scoring methods.

When we applied the PCA analysis of the consensus samples to the partial/non-consensus samples, the hybrid PCA demonstrated that a significant proportion of the partial consensus samples also segregated based on the majority diagnosis of the sample. Therefore, these four proteins together in conjunction with histologic analysis, are a promising panel that may more accurately classify BE samples between ND-BE/LGD and HGD/EAC with high sensitivity and specificity.

Some of these markers have been studied in dysplastic processes in other organ systems as well. For example, upregulation of p120ctn was seen in 37 % of gastric dysplasia cases and 66 % of gastric carcinoma cases in one study, sometimes associated with reduced membranous expression [31]. Also, c-myc copy number gain has been found to play a key role in the process of disease progression in cervical dysplasia [32, 33]. CDX2 staining has shown varying results in gastric neoplasia. CDX2 was not detected in normal gastric mucosa, while some studies have shown that CDX2 is associated with gastric epithelial dysplasia in 44–87 % of cases and CDX2 expression was also seen to gradually decrease from gastric epithelial dysplasia to early and advanced gastric cancers. However, these studies didn’t find any relationship between CDX2 expression and the degree of dysplasia [34, 35]. On the contrary, another study has shown that CDX2 expression was progressively reduced in gastric dysplasia as well as cancer [36]. However, most importantly none of these markers has been validated to be used in routine clinical practice for diagnosis or grading of dysplasia in these other organ systems.

While our study incorporates the expertise of three sub-specialized GI pathologists and uses a four-marker protein panel to test both consensus and non-consensus patient samples, we acknowledge that this is an initial study testing the utility of these biomarkers. Further validation will be needed before this protein panel can be used clinically, including prospective larger randomized studies involving larger patient cohorts from diverse locations with clinically relevant end points. Additionally, the IHC protocol used in this study will need to be streamlined with the use of clinically validated IHC reagents and conditions before it could be applied in routine clinical practice. However, we believe that the initial results in this study are very promising and this panel has future potential to serve as a useful adjunct for those challenging cases where the pathologist is struggling with the accurate diagnosis, which may ultimately affect optimal patient management.

A strength of this study is that two different semi-quantitative IHC scoring methods were evaluated in an attempt to determine the most clinically useful and accurate way of scoring the four-marker panel for BE. We found that the quartile scoring method had less sensitivity and specificity as well as a lower area under the curve, as compared to the IRS scoring. While scoring staining intensity may not currently be the most conventional way of IHC scoring in routine clinical practice, it appears with this panel of proteins, taking into account both the percentage and intensity of positively stained cells could lead to a more accurate diagnosis, particularly in those diagnostically challenging cases. Therefore, IRS scoring appears to be the better choice for the use of this four-marker protein panel and its use in grading BE dysplasia. Having said that, the sensitivity and specificity and/or area under the ROC curve of the quartile scoring method was still found to be more than many prior studies reported in the literature that have used different biomarkers [37–39].

A limitation of this study is the lack of progression data for ND-BE and LGD patients. While our sample set robustly segregates ND-BE and LGD from HGD and EAC, there are several samples from each group that segregate with the opposite group, although none of those are EAC. EAC samples completely segregate to the left of the PCA plot. It is intriguing to speculate that the biologic behavior of these samples may not match that of its histology. Would the three HGD samples that segregate with ND-BE and LGD samples be less likely to progress than the majority of HGD samples? Conversely, would the four ND-BE (*n* = 3) and LGD (*n* = 1) samples clustering with HGD and EAC be more likely to progress than most ND-BE and LGD? Since HGD is typically ablated, progression data would not be possible for those patients. This study, unfortunately, was a de-identified sample-set without patient outcomes and therefore these questions will need to be addressed in further studies that contain patient outcome.

Another limitation of this study is that this panel of biomarkers could not segregate ND-BE from LGD, and given that some institutions are choosing to actively treat LGD (either by ablation or endoscopic mucosal resection), this panel may not be a good diagnostic adjunct in challenging cases where the differential is between ND-BE and LGD. Despite this panel of biomarkers being unable to distinguish one major clinical decision point (no dysplasia versus dysplasia), it is effective at another decision point – LGD vs HGD. Given that some institutions are still treating LGD (surveillance) differently than HGD (with ablation and/or endoscopic resection), this may be the more crucial clinical decision point and our biomarker panel has utility in this decision.

Indefinite for dysplasia was deliberately excluded from this study, particularly because this study aimed to create and test a diagnostic panel that can be used as an adjunct to aid in more accurate grading of dysplasia in BE. Therefore, we wanted to use cases which were either definitively negative or positive for dysplasia. Further studies would be needed to evaluate this panel of markers in the “indefinite” category.

While a few markers have been evaluated previously for their potential as BE diagnostic markers, none have performed as well as the IHC panel presented here. The area under the curve generated by ROC analysis ranged between 0.607 and 0.852 in prior studies [37–39]. Using our own combination of markers, the areas under the ROC curve are 0.86 and 0.956 for the quartile scoring method and IRS scoring method, respectively, indicating a more accurate diagnosis of the disease than previous studies. Recently, AMACR (α-methylacyl coenzyme A racemase), an enzyme involved in β-oxidation of branched-chain fatty acids, has been shown to be a useful marker for differentiating ND-BE, LGD and HGD from each other, suggesting that AMACR might be a useful diagnostic discriminator [40, 41]. In a large series, AMACR was negative in all ND-BE cases, while it was positive in LGD cases (38 %), HGD cases (81 %) and EAC (72 %) [40]. However, the low sensitivity (less than 70 %) is a pitfall and does not allow a clinical use of AMACR [42]. Tp53 was also analyzed because of its association with dysplasia, though its staining has high false-positive and false-negative rates [43, 44]. However, Tp53 was never used in BE diagnosis because of the low specificity and sensitivity results observed in the IHC staining [45, 46]. Noticeably, in our study, the combinatorial approach using the above four biomarkers could segregate the samples into ND-BE/LGD versus HGD/EAC categories with both high sensitivity and specificity, although we acknowledge that these markers may not be helpful to differentiate ND-BE from LGD or HGD from EAC.

Interestingly, few studies have examined using a panel of markers for BE progression or staging of the disease. Bird-Lieberman *et al*. identified a panel of seven biomarkers analyzed in a population-based study that increases the accuracy of the prediction compared with any individual marker [47]. It would be interesting to evaluate whether these previously reported markers could increase the accuracy of the diagnosis when combined with our own panel. Nonetheless, we have shown that a combinatorial morphologic/IHC approach could successfully stratify patients into ND-BE/LGD and HGD/EAC categories and has the potential to optimize patient care. To date, this biomarker panel offers the greatest promise for grading Barrett’s esophagus based on ROC scores. Although histologic assessment still remains the gold standard for diagnosis of BE dysplasia, we propose that these proteins may serve as a useful assessment adjunct in the future, to be used in cases that are histologically challenging and where disagreement exists about the grade of BE dysplasia.

## Conclusions

In this initial study, we have identified a panel of four proteins, CDX2, p120ctn, c-Myc and Jagged1, that may be useful as an adjunct method of grading BE dysplasia. This panel of proteins is able to segregate ND-BE/LGD and HGD/EAC, distinguishing between LGD and HGD with high sensitivity and specificity. We propose that this IHC panel may be useful in discerning histologically challenging cases and may able to aid in the accurate diagnosis and management of patients. These promising results are preliminary findings, and therefore, further validation is required before the panel can be applied in routine clinical practice.

## Additional files

Additional file 1: Figure S1. Expression levels of four-marker protein panel in Barrett’s esophagus disease progression. (A) CDX2 expression with the quartile scoring method and (B) IRS scoring, decreased from ND-BE to EAC. (C) Membranous p120ctn expression with the quartile scoring method and (D) IRS scoring, decreased from ND-BE to EAC. (E) Cytoplasmic p120ctn expression with the quartile scoring method and (F) IRS scoring, increased from ND-BE to EAC. (G) c-Myc expression with the quartile scoring method and (H) IRS scoring, increased from ND-BE to EAC. (I) Jagged1 expression with the quartile scoring method and (J) IRS scoring. (JPG 156 kb) Additional file 2: Figure S2. Tp53 expression in partial consensus low-grade dysplasia cases. (A) Partial consensus low-grade dysplasia sample exhibiting positive staining for Tp53. (B) Partial consensus low-grade dysplasia sample exhibiting negative Tp53 staining. (a&b, 400×). (JPG 1 mb)

## Abbreviations

AMACR: α-methylacyl coenzyme A racemase
BE: Barrett’s esophagus
EAC: esophageal adenocarcinoma
GI: gastrointestinal
H&E: hematoxylin & eosin
HGD: high-grade dysplasia
HIPAA: health insurance portability and accountability act
IHC: immunohistochemistry
IMC: intramucosal esophageal adenocarcinoma
IRS: immunoreactivity score
LGD: low-grade dysplasia
ND-BE: non-dysplastic Barrett’s esophagus
p120ctn: p120-catenin
PCA: principal component analysis
ROC: receiver operating characteristic
SMC: submucosal esophageal adenocarcinoma
