# Supplementary figures and images for: Increasing diagnostic accuracy to grade dysplasia in Barrett’s esophagus using an immunohistochemical panel for CDX2, p120ctn, c-Myc and Jagged1
Source: Diagn Pathol. 2016 Feb 29;11:23. doi: 10.1186/s13000-016-0473-7 (PMC4772649; doi:10.1186/s13000-016-0473-7)

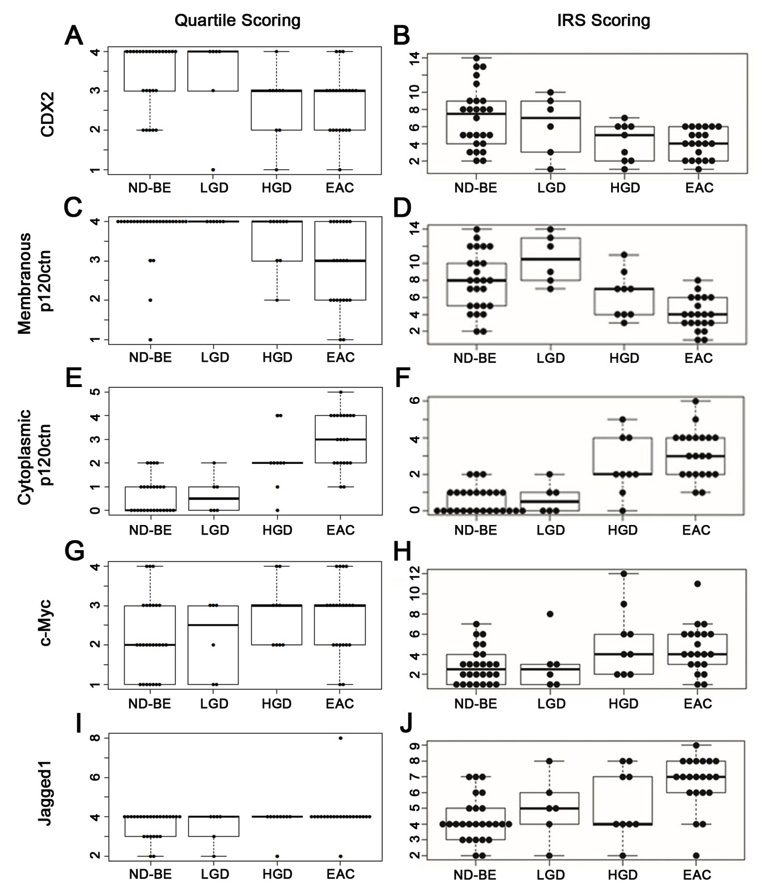

Supplement: Additional file 1: Figure S1. — Expression levels of four-marker protein panel in Barrett’s esophagus disease progression. (A) CDX2 expression with the quartile scoring method and (B) IRS scoring, decreased from ND-BE to EAC. (C) Membranous p120ctn expression with the quartile scoring method and (D) IRS scoring, decreased from ND-BE to EAC. (E) Cytoplasmic p120ctn expression with the quartile scoring method and (F) IRS scoring, increased from ND-BE to EAC. (G) c-Myc expression with the quartile scoring method and (H) IRS scoring, increased from ND-BE to EAC. (I) Jagged1 expression with the quartile scoring method and (J) IRS scoring. (JPG 156 kb) [file 13000_2016_473_MOESM1_ESM.jpg]

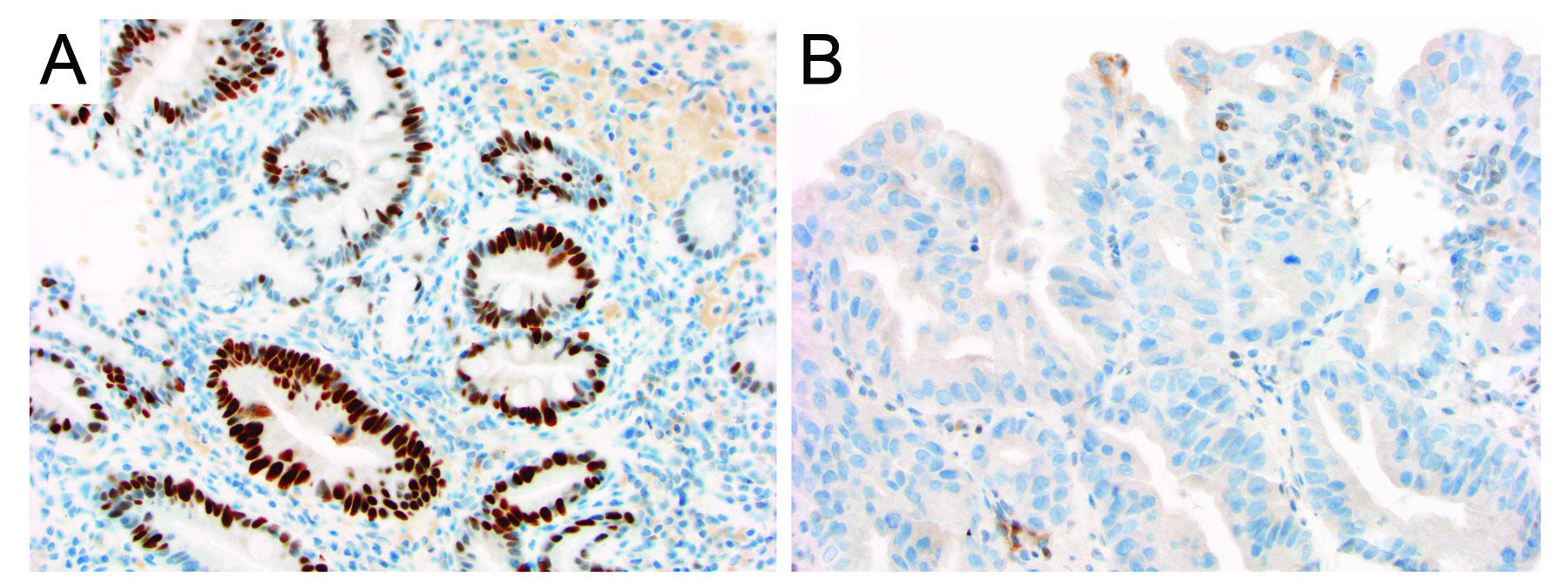

Supplement: Additional file 2: Figure S2. — Tp53 expression in partial consensus low-grade dysplasia cases. (A) Partial consensus low-grade dysplasia sample exhibiting positive staining for Tp53. (B) Partial consensus low-grade dysplasia sample exhibiting negative Tp53 staining. (a&b, 400×). (JPG 1 mb) [file 13000_2016_473_MOESM2_ESM.jpg]
